# The diagnostic and prognostic utility of repetitive nerve stimulation in patients with myasthenia gravis

**DOI:** 10.1038/s41598-023-30154-5

**Published:** 2023-02-20

**Authors:** Matthias Tomschik, Eva Renaud, Fiona Jäger, Chiara Paternostro, Jakob Rath, Walter Rinner, Gudrun Zulehner, Fritz Zimprich, Hakan Cetin

**Affiliations:** 1 Department of Neurosurgery, Medical University of Vienna, Vienna, Austria; 2 Department of Neurology, Medical University of Vienna, Vienna, Austria; 3 Comprehensive Center for Clinical Neurosciences and Mental Health, Medical University of Vienna, Vienna, Austria

**Keywords:** Neuromuscular disease, Prognostic markers, Diagnostic markers

## Abstract

Repetitive nerve stimulation (RNS) is a standard test for the diagnosis of myasthenia gravis (MG), where decrement of compound muscle action potentials (CMAP) corresponds to clinical muscle fatigability. Our aim was to ascertain the diagnostic and prognostic utility of RNS in MG patients. This study included MG patients treated between 01/2000 and 12/2016, with an observational period of at least one year and a minimum of two neurological examinations. Clinical and electrophysiological data were retrospectively gathered from patient records, and CMAP decrement was correlated with autoantibody titers and clinical disease severity at different time points. Ninety-four patients were included, with 88.3% of the cohort testing positive for acetylcholine receptor autoantibodies (AChR-Abs). RNS sensitivity was higher in patients with generalized disease (71.6%) than in purely ocular MG (38.5%). CMAP decrement did not significantly correlate with AChR-Ab titers, nor with clinical symptom severity at the time of testing or last follow up. However, there was a significant correlation between CMAP decrement and the worst recorded clinical status on a group level. RNS testing is more sensitive in generalized disease and AChR-Ab positive patients, but our data do not support RNS as a tool for long-term outcome prediction. Future studies with a prospective study design could help to overcome a number of limiting factors discussed in our study.

## Introduction

Myasthenia gravis (MG) is an autoimmune disorder of the neuromuscular junction with autoantibodies directed against the muscle-type acetylcholine receptor (AChR) at the postsynaptic membrane in the majority of patients. Other antigens identified to be targeted by autoantibodies in MG are the muscle specific kinase (MuSK) and low-density lipoprotein receptor-related protein 4 (LRP4)^1^. In yet another group of MG patients, no autoantibodies can be detected using common diagnostic assays, which therefore is classified as seronegative MG (SNMG)^2^. Clinically, all forms are characterized by increased fatigability and weakness of ocular, bulbar and/or limb muscles. The disease course is highly variable with long periods of minimal manifestation in some patients and frequent exacerbations of muscle weakness in others^3^. A wide range of different therapies is available especially for MG patients with severe symptoms, the early identification of which could help to optimize the long-term outcome. Currently patients are stratified based on clinical and serological characteristics^2,4–6^, but there is a substantial need to establish prognostic tools that help to better identify patients at risk for disease exacerbations.

Repetitive nerve stimulation (RNS) has been established as a diagnostic mainstay in MG, although its sensitivity was reported to be highly variable and dependent on disease severity^7^ and the distribution of affected muscles^8^. In a more recent report, a pathologic decrement in limb muscles has also been found to be predictive of symptom generalization in ocular MG patients^9^, revealing RNS as a potential prognostic tool in MG. However, there is insufficient evidence to further underpin the prognostic significance of RNS. In this project, we therefore aimed to assess the diagnostic and prognostic utility of RNS in MG patients.

## Methods

### Ethical approval

This study was approved by the Ethical Committee of the Medical University of Vienna (EK 2062/2018), with a waiver of the requirement for informed consent because of the retrospective design. All of the research described was performed in accordance with the Declaration of Helsinki and in line with Good Scientific Practice guidelines.

### Study design and patient ascertainment

This retrospective single-center cohort study included MG patients treated at the Department of Neurology, Medical University of Vienna, a tertiary care center, between January 2000 and December 2016. Patients’ charts were reviewed by a neurologist with neuromuscular specialization. Diagnosis of MG was based on the presence of characteristic symptoms together with increased levels of autoantibodies against AChRs or MuSK, or in the case of SNMG patients, on a clear response to acetylcholine esterase inhibitor treatment. All patients in the cohort were tested for AChR autoantibodies using a radioimmunoassay (RIA) or MuSK autoantibodies using enzyme linke immunosorbent assays (ELISA) at one point during their disease course. An observational period of at least one year with a minimum of two neurological examinations were required for patients to be included, with the initial neurological examination performed within seven days from the date of RNS testing. Patients were considered to have an ocular onset when symptoms were limited to the eyes for the first three months, which was regarded the disease onset period. Disease severity was graded according to the Myasthenia Gravis Foundation of America (MFGA) classification, and the outcome was assessed using the MGFA post-intervention status (MGFA-PIS). Patients fulfilling the criteria for complete stable remission, pharmacologic remission and minimal manifestation status were grouped as “asymptomatic”, which is generally considered as the treatment goal in MG^10^. The outcome at the last recorded visit was further dichotomized into optimal clinical outcome (MGFA-PIS of minimal manifestation status or better) and non-optimal outcome (detectable weakness).

### Repetitive nerve stimulation

Repetitive nerve stimulation was performed by a certified biomedical technician and interpreted by a neurologist with neuromuscular specialization. Patients were advised to stop pyridostigmine treatment for at least six hours before RNS testing. Trains of six square wave pulses were applied through bipolar stimulation electrodes at a frequency of 3 Hz and surface recording electrodes were placed so that supramaximal nerve stimulation produced an initial sharp negative deflection of the compound muscle action potential (CMAP). Muscles for RNS testing were generally selected according to clinical weakness and fatigability and included one facial and one proximal upper extremity muscle in most patients. Decrement was defined as the ratio between the first and either the fourth or fifth CMAP amplitude, depending on which was lower. A decrement of over 10% in at least one muscle was judged a pathologic result.

### Statistics

Differences were analyzed for significance using Fisher’s exact test for categorical variables, and Mann–Whitney-U test for continuous variables. The correlation between clinical states on an ordinal scale (MGFA classification) and the RNS decrement was analyzed with Kendall’s Tau coefficient. For this exploratory analysis two-sided *p*-values < 0.05 were considered significant. Statistical analyses and plotting of data were performed using R version 3.4.3 (www.r-project.org).

## Results

### Cohort characteristics

We identified 117 patients with MG who underwent RNS testing at the Department of Neurology, Medical University of Vienna, during the specified study period. Of these, 4 patients were excluded due to insufficient description of their clinical state at the time of testing and another 19 patients due to insufficient follow-up. We were thus able to include 94 MG patients with a median age of 61.8 years (ranging from 17.5 to 87.7 years) and females accounting for 53.2% of the cohort (Table 1). Autoantibodies against the AChR (AChR-Ab) could be detected in 83 patients (88.3%), autoantibodies against MuSK (MuSK-Ab) in three patients (3.2%) and eight patients (8.5%) were classified as SNMG. Twenty-nine patients (30.9%) had exclusively ocular symptoms (MGFA I) at onset (Table 1) with 16 patients secondarily generalizing during the disease course after a median of 30.4 weeks (IQR 17.4–54-7). All three MuSK-Ab positive patients had a generalized onset and in the SNMG group an ocular onset was found in 50% of the patients. Sixty-two patients (66.0%) exhibited their worst clinical status at disease onset with the remainder undergoing clinical worsening during the following course of their disease.

**Table 1:** Description of the entire study cohort and the AChR-Ab positive patients.

	All patients	AChR-Ab positive
	*N* = 94	*N* = 83
Median age (IQR)	61.8 (36.2–71.7)	63.3 (40.4–72.7)
Sex (%)		
Female	50 (53.2)	42 (50.6)
Male	44 (46.8)	41 (49.4)
AChR antibody concentration, nmol/L (± SD)	–	41.7 (± 71.4)
Subgroups according to^2^ (%)		
Early onset MG	26 (27.7)	26 (31.3)
Late onset MG	51 (54.3)	51 (61.4)
MuSK-Ab positive	3 (3.2)	0
SNMG	8 (8.5)	0
Thymoma associated MG	6 (6.4)	6 (7.2)
Median diagnostic latency in days (IQR)	30 (109.0)	30 (110.5)
MGFA class at disease onset (%)		
I	29 (30.9)	25 (30.1)
II	53 (56.4)	48 (57.8)
III	7 (7.4)	5 (6.0)
IV	4 (4.3)	4 (4.8)
V	1 (1.1)	1 (1.2)

### Diagnostic yield of RNS

**Figure 1:**
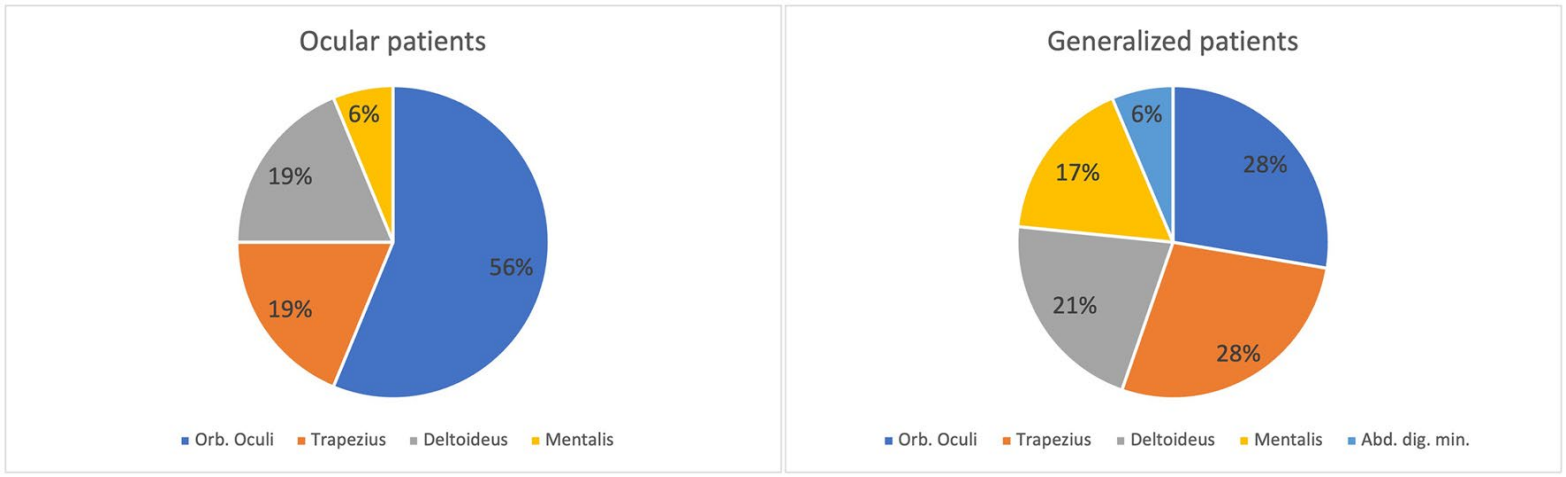
Frequency of muscles with highest CMAP decrement: While the orbicularis oculi muscle was the most affected muscle on RNS testing in ocular patients, the distribution was more even in generalized patients.

A total of 94 patients with MG were tested in our cohort with a mean of 1.96 muscles tested per patient. Median times from symptom onset to RNS testing and from clinical diagnosis to RNS testing were 63.5 (interquartile range: 15.5–157.75) and 2.5 (interquartile range: 0–26.5) days, respectively. At the time of RNS testing, six patients were using prednisolone, one azathioprine and one both prednisolone and azathioprine, totaling 8 patients (8.5%) in our cohort. The most commonly examined muscles were the orbicularis oculi (n = 66, 70.2%), the trapezius (n = 47, 50.0%), the mentalis (n = 35, 37.2%), the deltoid (n = 23, 24.3%), and the abductor digiti minimi muscle (n = 11, 11.7%). In patients with ocular symptoms only, facial muscles were tested in all patients and limb muscles in 56.3% of this group. In patients with generalized symptoms, by contrast, the frequency of RNS in limb and facial muscles was comparable (83.9% and 79.0%, respectively). The most affected muscle in patients with ocular symptoms only was the orbicularis oculi muscle (56.3%), while the picture was less clear in patients with generalized symptoms. In these patients the orbicularis oculi, trapezius, and deltoid muscles were most severely affected with a pathologic decrement in 27.7%, 27.7% and 21.3%, respectively (Fig. 1).

RNS sensitivity was higher in primarily and secondarily generalized disease (58/81 patients, 71.6%) as compared to patients with purely ocular symptoms (5/13 patients, 38.5%), but we found no difference between primarily and secondarily generalized disease. All three MuSK-Ab positive patients had a pathologic decrement on RNS testing, while only 67.5% of patients with AChR-Ab and 50% of SNMG patients showed a pathologic CMAP decrement. In patients with a pathologic RNS result, mean decrement was 30.7 ± 17.3%).

### Correlation between AChR-Ab concentrations and CMAP decrement

**Figure 2:**
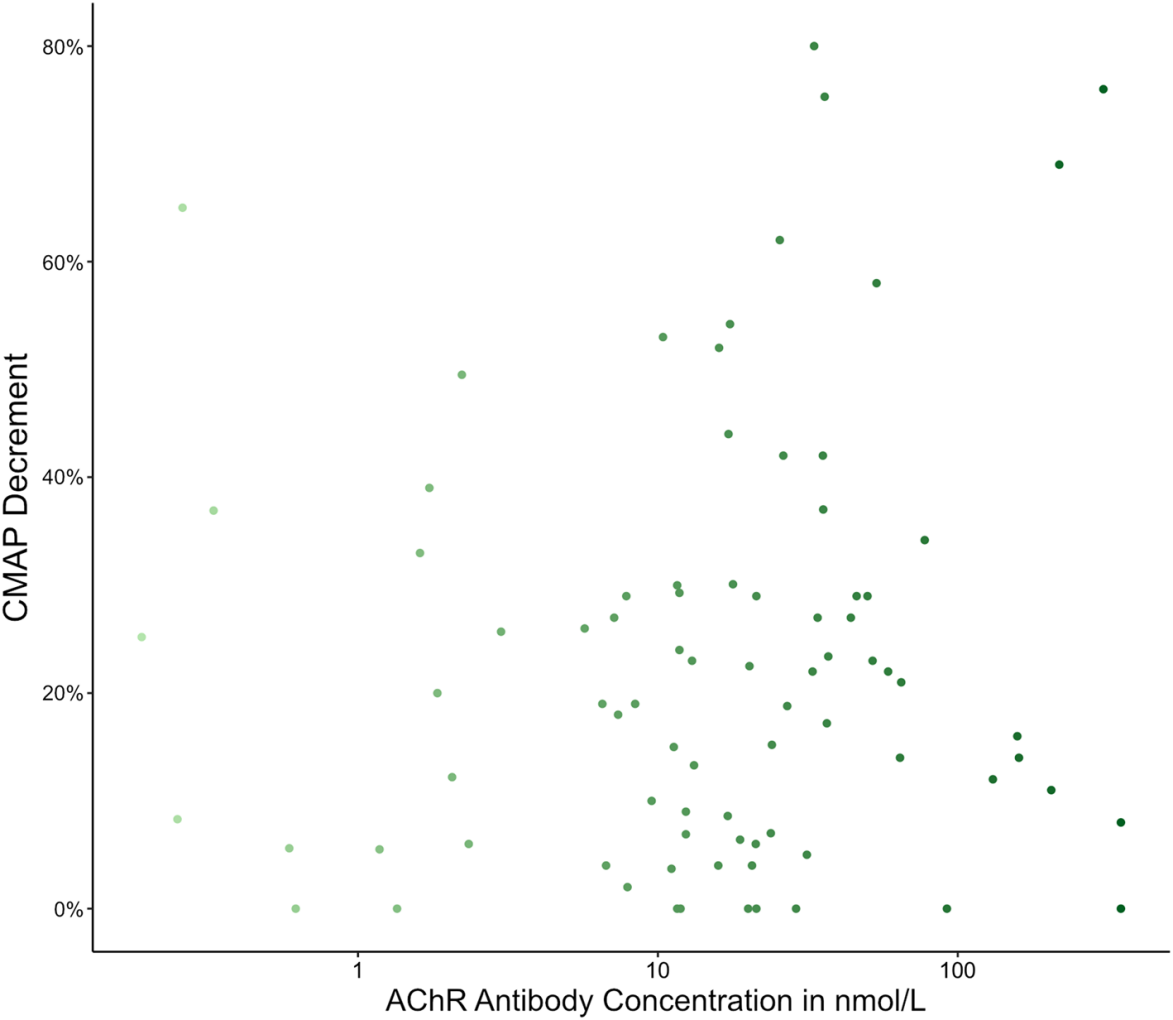
RNS-Antibody-Correlation: There is no clear correlation of CMAP decrement at RNS testing with the concentration of AChR antibodies in patients with AChR-Ab positive MG disease. The x-axis is represented on a logarithmic scale for better visualization.

The mean AChR-Ab titer was 41.7 nmol/L (SD ± 71.4) with a median time between serum AChR-Ab testing and RNS of 0 days (IQR 0–9 days). There was no correlation between AChR-Ab concentrations and CMAP decrement on RNS testing (Pearson’s R = − 0.079, *p* = 0.482; Fig. 2). However, AChR-Ab concentrations correlated with clinical status as defined by MGFA classification at onset (Kendell’s tau = 0.18, *p* = 0.033), and a purely ocular MG manifestation was associated with lower AChR-Ab concentrations as compared to patients with generalized MG (7.5 ± 8.6 nmol/L [n = 32] vs. 46.0 ± 74.6 nmol/L [n = 62], *p* = 0.005).

### Correlation between symptom severity and CMAP decrement

**Figure 3:**
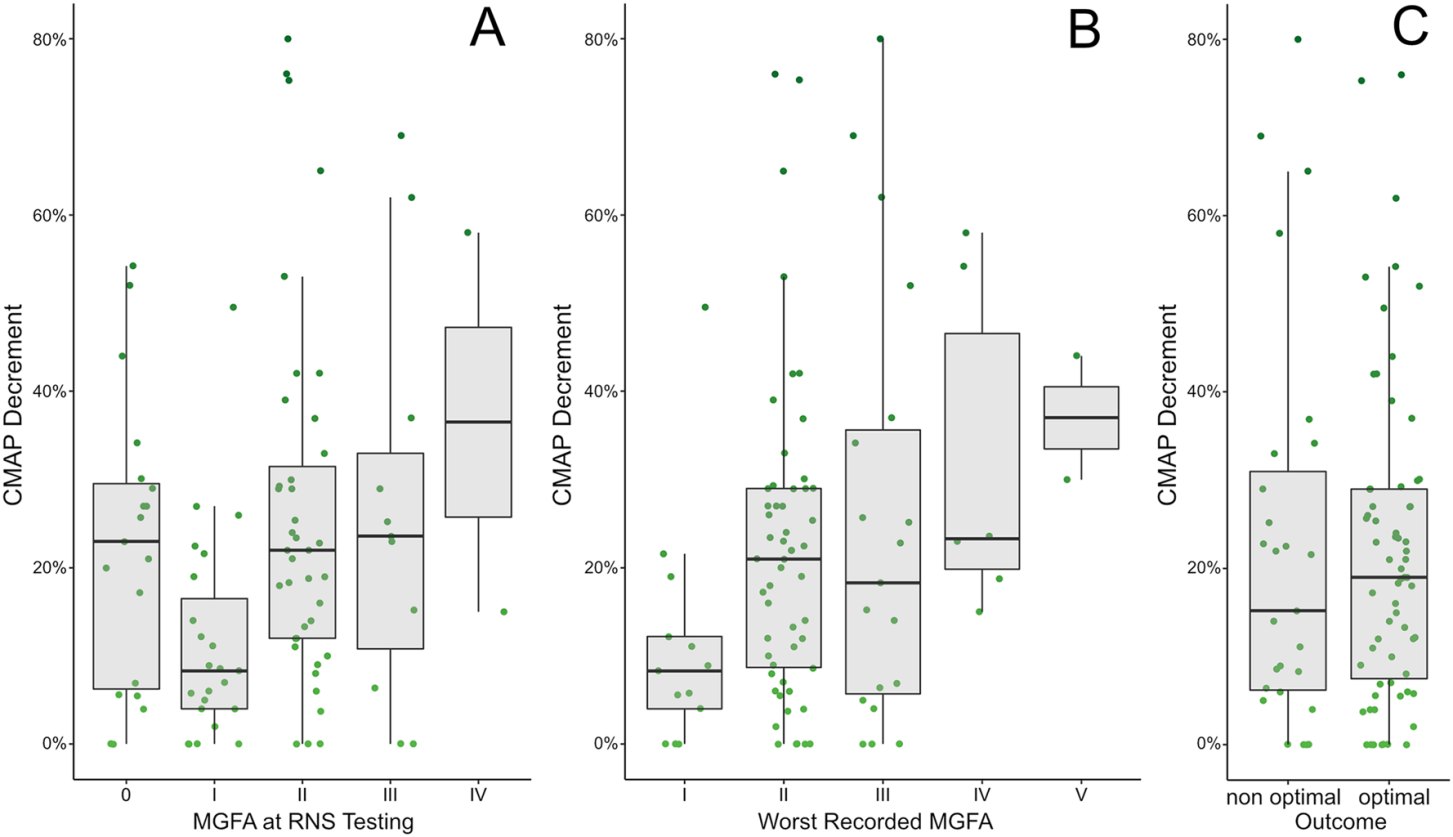
Correlation of CMAP decrement and clinical state: Data are presented on a group level (boxplot) and for each individual patient (green dots). (**A**) No clear correlation between clinical severity at testing and CMAP decrement exists, mainly because clinically asymptomatic patients still exhibit pathologic decrements at RNS (**B**) Correlation of CMAP decrement with the worst recorded MGFA is evident on the group level, but patients with the high CMAP decrement did not necessarily develop severe disease or myasthenic crisis during the observed period. (**C**) There is no association of CMAP decrement with the outcome of patients at last follow-up.

Patients had a mean observation period of 2.4 ± 1.5 years and were periodically assessed for symptom severity and treatment response. In our cohort, CMAP decrement was lower in ocular MG patients as compared to patients with primarily or secondarily generalized disease (11.2 ± 13.4% vs. 23.6 ± 19.4%, *p* = 0.010), but in patients with ocular onset there was no difference between those who would later generalize and those who would remain purely ocular (*p* = 0.129). CMAP decrement also correlated significantly with the clinical status graded by the MGFA classification at onset (Kendall’s tau = − 0.226, *p* = 0.006) but not with clinical status around the time of RNS testing (Kendall’s tau = − 0.134, *p* = 0.090, Fig. 3A). There was however a significant correlation between worst clinical status and CMAP decrement on RNS (Kendall’s tau = − 0.21, *p* = 0.010), although there was also a number of patients with a mild disease course despite very high CMAP decrements > 50% (Fig. 3B).

At their last respective follow up visit, 67 patients (71.3%) were clinically asymptomatic (as defined by a MGFA-PIS of complete stable remission, pharmacological remission, and minimal manifestation), 8 patients exclusively had ocular symptoms (8.5%), while 14 (14.9%) and 5 patients (5.3%) were characterized by generalized MG symptoms corresponding to MGFA classes II and III, respectively. Ultimately, we found no correlation between CMAP decrement on RNS testing with clinical status at last follow up (Kendall’s tau = 0.03, *p* = 0.711). Dichotomization of the outcome into optimal outcome (MGFA-PIS of minimal manifestation status or better) and non-optimal outcome did not change this finding (*p* = 0.725; Fig. 3C).

## Discussion

In this single-center cohort study, we present data on the diagnostic and prognostic utility of RNS including test sensitivity calculations and correlations between CMAP decrement and clinical outcome measures. Test sensitivity has been the subject of multiple previous publications, but the reported results vary greatly between studies and depend on various factors including disease severity, MG subform^8,11–13^ and the patients’ antibody status. AChR- and MuSK-Ab positive patients were shown to be more frequently diagnosed by RNS as compared to double-seronegative patients^14^, with pooled sensitivities of 29% and 79% for ocular and generalized MG, respectively^15^, which are close to our sensitivity figures of 38.5% in ocular MG and 71.6% in generalized MG. In our study, the orbicularis oculi was among the most sensitive muscles in ocular as well as generalized MG, which is in line with previous findings^11,16^. However, RNS sensitivity was shown to increase with the number of tested muscles, and, as a result, the bilateral examination of three muscles including the orbicularis oculi, anconeus and trapezius muscles were suggested to be tested to maximize sensitivity^11^.

Another aim of the present study was to correlate CMAP decrement with clinical outcome measures at different time points to determine the prognostic significance of RNS. We found significant correlations of CMAP decrement with disease severity at onset and with maximum disease severity during the observed period, which is in line with a previous study reporting associations of higher CMAP decrement with generalized disease and higher quantitative myasthenia gravis scores^7^. We found a correlation between CMAP decrement and the worst recorded clinical status on a group level, but according to our data not strong enough on an individual level to be clinically useful. A direct correlation with the clinical state at the time of testing is however more difficult. Our results did not show a strong association between CMAP decrement at RNS and graded clinical severity, as reported before in another study, where no correlation between amplitude decrement in the deltoid muscle and a patient-derived outcome score could be detected^17^. The lack of a statistical correlation between CMAP decrement and MGFA scores at the time of testing in our study was most likely due to asymptomatic patients under immunosuppressive treatment still having highly pathologic CMAP decrements. Interestingly, another group found a correlation between quantitative MG scores and RNS decrement^18^, but following the initiation of treatment no correlation between CMAP decrement changes and treatment response could be found^19^. Moreover, we could not identify significant differences in ocular patients who remained ocular during the disease course and ocular patients with secondary generalization, which is in contrast to a previous study, where a pathologic CMAP decrement in limb muscles of ocular patients was found to be predictive of symptom generalization^9^. Our findings therefore do not underpin a significant role of RNS for MG prognosis. Single fiber electromyography, by contrast, could be more useful for the prediction of MG prognosis, as changes in consecutive jitter measurements were shown to significantly correlate with clinical outcome measures^20^. Future studies should assess the role of consecutive electrophysiological testing for the prediction of MG prognosis or as a warning for clinical deterioration. Objective and quantifiable information about when treatments should be escalated or could be deescalated, would be tremendously useful.

There are some limitations related to the retrospective methodology of this study to be noted. The follow-up period was not standardized and varied between individual patients, but as fluctuations in disease severity are a hallmark of MG and can even occur in later disease course^6^, the varying follow-up times are unlikely to have biased the results. Another limitation is the lack of standardized muscle testing. We routinely evaluate muscles that are clinically affected, which again varies from patient to patient. This could have led to an underestimation of clinically unaffected muscles with a pathologic CMAP decrement and therefore might have influenced the analysis on a potential role of RNS in ocular patients to predict symptom generalization. Moreover, detailed information on weakness of specific muscle groups (e.g., graded according to the Medical Research Council Scale) at the time of RNS testing and during the disease course was not available and could therefore not be correlated with CMAP decrement, which could have provided a more precise measure of how RNS results correlate with clinical strength. RNS results could also have been influenced by the use of pyridostigmine or other immunosuppressive drugs around the time of RNS testing. To overcome this limitation, patients were instructed not to use pyridostigmine at least six hours before RNS testing, but this interval might have been too short especially in patients with milder symptoms. Prednisolone and azathioprine, by contrast, were only used by a few patients for short periods, and are therefore unlikely to have biased the results. And finally, while our sample size was comparable to other studies, the inclusion of larger cohorts might be required to determine the prognostic significance of RNS more accurately.

## Conclusion

Our data indicate that RNS is more sensitive in generalized than in ocular MG with its overall sensitivity being less than desirable. Especially in patients without detectable autoantibodies the diagnostic accuracy is decreased. While there is a correlation with disease severity at onset, CMAP decrement does not significantly correlate with antibody concentrations in AChR-Ab positive patients, the clinical severity at the time of testing, nor with the clinical outcome at last follow-up. Therefore, our data do not support RNS as a tool for long-term outcome prediction, limiting the clinical use of RNS to diagnostic purposes. In the era of highly sensitive assays for the detection of specific MG autoantibodies^21,22^, however, the clinical use of RNS might become limited to SNMG cases to confirm diagnosis, where a sufficient number of muscles need to be tested to maximize sensitivity.

## Data Availability

Pseudonymized patient data, including clinical and electrophysiological details can be shared on reasonable request to the corresponding author after approval by an accredited ethics committee and the data clearing commission of the Medical University of Vienna.

## References

[1] Cetin H, Vincent A (2018). Pathogenic mechanisms and clinical correlations in autoimmune myasthenic syndromes. Semin. Neurol..

[2] Gilhus NE, Verschuuren JJ (2015). Myasthenia gravis: Subgroup classification and therapeutic strategies. Lancet. Neurol..

[3] Neumann B (2020). Myasthenic crisis demanding mechanical ventilation: A multicenter analysis of 250 cases. Neurology.

[4] Beghi E (1991). Prognosis of myasthenia gravis: A multicenter follow-up study of 844 patients. J. Neurol. Sci..

[5] Cortés-Vicente E (2020). Clinical and therapeutic features of myasthenia gravis in adults based on age at onset. Neurology.

[6] Tomschik M (2020). Subgroup stratification and outcome in recently diagnosed generalized myasthenia gravis. Neurology.

[7] Abraham A (2017). Electrophysiological testing is correlated with myasthenia gravis severity. Muscle Nerve.

[8] Costa J, Evangelista T, Conceição I, de Carvalho M (2004). Repetitive nerve stimulation in myasthenia gravis–relative sensitivity of different muscles. Clin. Neurophysiol. Off. J. Int. Feder. Clin. Neurophysiol..

[9] Kim KH, Kim SW, Shin HY (2021). Initial repetitive nerve stimulation test predicts conversion of ocular myasthenia gravis to generalized myasthenia gravis. J. Clin. Neurol..

[10] Sanders DB (2016). International consensus guidance for management of myasthenia gravis: Executive summary. Neurology.

[11] Bou Ali H (2017). New strategy for improving the diagnostic sensitivity of repetitive nerve stimulation in myasthenia gravis. Muscle Nerve.

[12] Kennett RP, Fawcett PR (1993). Repetitive nerve stimulation of anconeus in the assessment of neuromuscular transmission disorders. Electroencephalogr. Clin. Neurophysiol..

[13] Zinman LH (2006). Sensitivity of repetitive facial-nerve stimulation in patients with myasthenia gravis. Muscle Nerve.

[14] Oh SJ (2006). Repetitive nerve stimulation of facial muscles in MuSK antibody-positive myasthenia gravis. Muscle Nerve.

[15] Benatar M (2006). A systematic review of diagnostic studies in myasthenia gravis. Neuromus. Disord. NMD.

[16] Zambelis T, Kokotis P, Karandreas N (2011). Repetitive nerve stimulation of facial and hypothenar muscles: Relative sensitivity in different myasthenia gravis subgroups. Eur. Neurol..

[17] Rostedt A, Padua L, Stålberg EV (2005). Correlation between a patient-derived functional questionnaire and abnormal neuromuscular transmission in Myasthenia Gravis patients. Clin. Neurophysiol. Off. J. Int. Feder. Clin. Neurophysiol..

[18] Barnett C, Katzberg H, Nabavi M, Bril V (2012). The quantitative myasthenia gravis score: Comparison with clinical, electrophysiological, and laboratory markers. J. Clin. Neuromuscul. Dis..

[19] Zinman L, Baryshnik D, Bril V (2008). Surrogate therapeutic outcome measures in patients with myasthenia gravis. Muscle Nerve.

[20] Sanders DB, Massey JM (2017). Does change in neuromuscular jitter predict or correlate with clinical change in MG?. Muscle Nerve.

[21] Rodriguez Cruz PM, Huda S, Lopez-Ruiz P, Vincent A (2015). Use of cell-based assays in myasthenia gravis and other antibody-mediated diseases. Exp. Neurol..

[22] Huda S (2017). IgG-specific cell-based assay detects potentially pathogenic MuSK-Abs in seronegative MG. Neurol. Neuroimmunol. Neuroinflamm..

